# A Novel Method for Digital Reconstruction of the Mucogingival Borderline in Optical Scans of Dental Plaster Casts

**DOI:** 10.3390/jcm11092383

**Published:** 2022-04-24

**Authors:** Leonard Simon Brandenburg, Stefan Schlager, Lara Sophie Harzig, David Steybe, René Marcel Rothweiler, Felix Burkhardt, Benedikt Christopher Spies, Joachim Georgii, Marc Christian Metzger

**Affiliations:** 1 Medical Center—University of Freiburg, Center for Dental Medicine, Department of Oral and Maxillofacial Surgery, Faculty of Medicine, University of Freiburg, Hugstetter Str. 55, 79106 Freiburg, Germany; stefan.schlager@anthropologie.uni-freiburg.de (S.S.); lara.sophie.harzig@uniklinik-freiburg.de (L.S.H.); david.steybe@uniklinik-freiburg.de (D.S.); rene.rothweiler@uniklinik-freiburg.de (R.M.R.); marc.metzger@uniklinik-freiburg.de (M.C.M.); 2 Medical Center—University of Freiburg, Center for Dental Medicine, Department of Prosthetic Dentistry, Faculty of Medicine, University of Freiburg, Hugstetter Str. 55, 79106 Freiburg, Germany; felix.burkhardt@uniklinik-freiburg.de (F.B.); benedikt.spies@uniklinik-freiburg.de (B.C.S.); 3 Fraunhofer Institute for Digital Medicine MEVIS, Bremen, Max-von-Laue-Str. 2, 28359 Bremen, Germany; joachim.georgii@mevis.fraunhofer.de

**Keywords:** statistical shape model, mucogingival borderline, implant planning, soft tissue dimensions, virtual planning

## Abstract

Adequate soft-tissue dimensions have been shown to be crucial for the long-term success of dental implants. To date, there is evidence that placement of dental implants should only be conducted in an area covered with attached gingiva. Modern implant planning software does not visualize soft-tissue dimensions. This study aims to calculate the course of the mucogingival borderline (MG-BL) using statistical shape models (SSM). Visualization of the MG-BL allows the practitioner to consider the soft tissue supply during implant planning. To deploy an SSM of the MG-BL, healthy individuals were examined and the intra-oral anatomy was captured using an intra-oral scanner (IOS). The empirical anatomical data was superimposed and analyzed by principal component analysis. Using a Leave-One-Out Cross Validation (LOOCV), the prediction of the SSM was compared with the original anatomy extracted from IOS. The median error for MG-BL reconstruction was 1.06 mm (0.49–2.15 mm) and 0.81 mm (0.38–1.54 mm) for the maxilla and mandible, respectively. While this method forgoes any technical work or additional patient examination, it represents an effective and digital method for the depiction of soft-tissue dimensions. To achieve clinical applicability, a higher number of datasets has to be implemented in the SSM.

## 1. Introduction

Osseo-integrated implants have emerged as the gold standard in dental care since their invention by Brånemark in the 1970s [1,2]. Highly aesthetic results, comfort in wear and the preservation of neighboring teeth describe only a few of the advantages that come with the use of implant-supported prostheses [3,4]. Unlike early implant systems, today’s modern implants achieve long-term survival rates and may last for decades, making them a reliable restorative option [5,6]. Moreover, sophisticated screening tools [7,8] help to identify patients suitable for implant treatment [9,10]. In addition to the continuous improvement of implant designs, pre-operative planning options have developed decisively to improve long-term stability of implants. Modern computer-tomography imaging [11] and the invention of computer-assisted surgery [12] allow advanced implant site assessment and pre-operative virtual planning [13,14,15,16,17,18]. Especially complex implant cases, e.g., patients with syndromal malformations [19,20,21], reduced mouth opening [22] or poor bone quality [23], require an impeccable preoperative planning to enable safe and successful implant placement. Modern planning software allows the determination of an ideal implant position that matches both the patient-specific anatomy and the requirements of the desired dental prosthesis [13,14,15,16,17,18]. Furthermore, pre-operative virtual planning increases the efficiency of the whole treatment procedure [24,25].

Current literature reports that pre-operative planning should consider soft-tissue dimensions to guarantee long-term success [26,27,28,29]. In fact, recent studies have shown that an adequate amount of attached gingiva around dental implants leads to increased hard- and soft-tissue stability [30,31], better esthetic results [32], reduced plaque accumulation and a lower incidence of peri-implant mucositis [33]. It can be stated that implants should only be placed in regions of the jaw covered with a sufficient amount of attached gingiva.

In healthy patients, the attached gingiva extends from the level of the marginal gingiva to the mucogingival borderline (MG-BL). The marginal gingiva is easily identifiable at the borderline between mucosa and tooth (referred to as mucous–tooth borderline, “MT-BL” in the following). The MG-BL describes the transition from attached to flexible mucosa [34]. This junction line becomes obscured by contact pressure on the gingiva during conventional impression taking. In contrast, intra-oral scans (IOS) can depict the MG-BL due to the difference in color appearance of attached gingiva and free mucosa. However, despite increasing digitalization in dentistry, in some cases the use of plaster casts is preferred, due to the haptic features of physical models or the lack of modern intra-oral scanners [35,36]. Recent review articles on dental implant planning still describe the use of plaster casts as the current state of the art [17]. It should be noted that scans of plaster models do not allow to identify the MG-BL when fed into the virtual planning process via digitization [16,17]. Considering the fundamental relevance of the attached gingiva for long-term success of dental implants, different approaches were proposed to reconstruct the MG-BL in plaster casts [37,38,39]. The aim of this study was to visualize the course of the MG-BL based on the information available from conventionally fabricated plaster casts using a statistical shape model (SSM), which processes empirical anatomical data. Moreover, the aim of this study was to assess if and to what degree the shape of MG-BL can be statistically modeled as a function of MT-BL. The working hypothesis can be stated as follows: it is possible to derive the morphology of the MG-BL of healthy individuals from geometric information provided by a digitized plaster cast using a digital workflow.

## 2. Materials and Methods

This study was approved by the ethics committee of the Albert-Ludwigs-University Freiburg, Germany (No. 251/20, renewed by No. 21/1451) and was registered in the German Clinical Trials Register (No. DRKS00027435). All participants gave written informed consent for study implementation.

Data processing, analyses and shape model creation were performed using the R-packages Morpho, Rvcg [40,41] and RvtkStatismo [42], which are available for the statistical/mathematical platform R [43].

### 2.1. Study Design

In this prospective study, conventional dental impressions, as well as IOS, were taken from dental students and employees of the Faculty of Dentistry of the University of Freiburg (see Section 2.3 and Section 2.4). Based on the acquired IOS, an SSM was generated, which captured the shape variability of the MG-BL and the MT-BL within the study sample. The plaster casts were scanned and saved as digital models. For each digitized model, the MG-BL was reconstructed using a temporarily created SSM in a Leave-One-Out Cross Validation (LOOCV) (see Section 2.5 and Section 2.6). Differences between the calculated and the original MG-BL were determined by comparison of the IOS with the SSM-based reconstruction to assess the accuracy of this workflow (see Section 2.7).

### 2.2. Inclusion and Exclusion Criteria

Dental medicine students and employees aged over 18 years at the Faculty of Dentistry of the University of Freiburg, who signed an informed consent, were considered for study implementation. The following inclusion criteria were defined for this study: adequate oral hygiene, no periodontal or gingival diseases and no existent dental implants. All participants had a continuous dental arch with existent teeth 17–27 and 37–47 (FDI-scheme).

Subjects under 18 years of age or with any type of periodontal or gingival disease, dental implants or missing teeth (except wisdom teeth) were excluded from the study.

### 2.3. Creation of IOS and Plaster Casts

**Figure 1:**
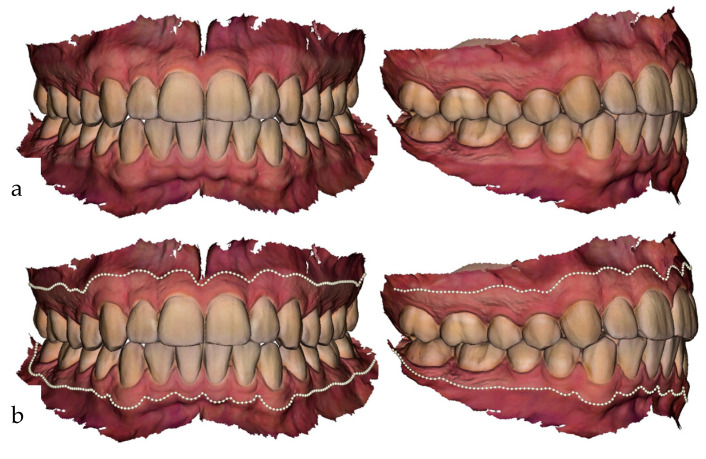
The MG-BL can be identified by the difference in color of attached gingiva and free mucosa. The MT-BL is clearly visible at the junction between tooth and gingiva (**a**). The MT-BL and MG-BL are placed in 3D Slicer using curves based on sliding semi-landmarks (**b**).

The IOS were performed using an intra-oral scanner (Trios 4, 3Shape, Copenhagen, Denmark). The scanning procedure was performed according to the manufacturer’s instructions, and the suggested scan paths were applied. Extent areas of the IOS were cropped if necessary and the mesh was exported as Polygon File (PLY), which includes color information. Plaster casts were produced using alginate impression material (Alginoplast^®^, Kulzer Mitsui Chemicals Group, Hanau, Germany) and dental stone (pico-crema soft^®^ type 3 DIN EN ISO 6873, Picodent, Wipperfürth, Germany). Digitization was performed using an optical scanner (E3, 3Shape), and the data was equally exported as PLY files (see Figure 1).

### 2.4. Preparation of the Data

**Figure 2:**
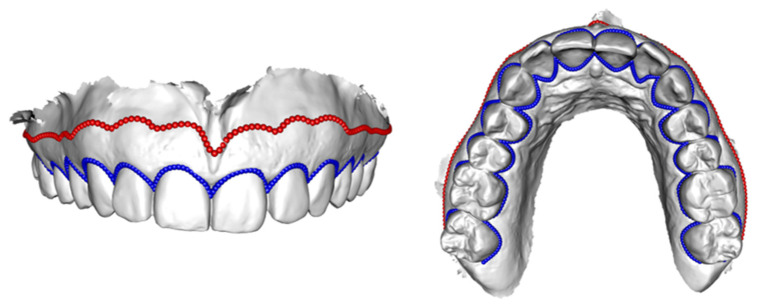
Detailed depiction of the sliding semi-landmarks set along the MT-BL (blue) and MG-BL (red). To achieve a proper representation of the MT-BL the tip of each interdental papilla was annotated with one additional landmark from each side (oral and buccal).

Two different curves, which indicate the course of the MT-BL and the MG-BL, were set on the IOS using the open-source software 3D Slicer [44] (see Figure 1b and Figure 2). The curves were set using sliding semi-landmarks [45] (see Figure 2). The MT-BL was set at the transition from mucous membrane to tooth using 560 semi-landmarks. The tip of the interdental papillae was marked additionally with one landmark from both buccal and oral side to capture the morphology of the MT-BL in detail. The MG-BL was set along the transition from attached to flexible mucosa using 150 semi-landmarks (see Figure 2). To minimize noise along the curves, landmarks were allowed to slide along the curves [45]. Furthermore, four identical anatomical landmarks were set on each of the surface scans and the corresponding digitized models for superimposition of the two surfaces later on.

### 2.5. Statistical Shape Model

Statistical shape modeling is a validated method for automatic processing of medical imaging data [46]. SSM are computed from a set of spatially registered individual shapes—e.g., anatomical structures—from which they “learn” the variability of valid shapes. SSM model that variability and parametrize the boundaries within which the shape is allowed to vary as a probability distribution [47]. Thus, in addition to the mere identification of anatomical structures, the shape of missing or damaged structures can be computed within medical imaging datasets using SSM [48,49,50,51,52]. The clinical use of SSM for creating digital dental wax-ups was demonstrated recently [53]. To extend the applicability of shape modeling in the field of implant planning, an SSM to predict the MG-BL based on the morphology of the MT-BL was generated. A separate SSM for the upper and lower jaw was deployed based on the combined landmark information annotated in the training data (see Figure 1). To build the SSM, the semi-landmarks were allowed to slide along the curves, minimizing Thin Plate Spline-bending energy, in order to remove noise associated with inconsistent digitization [45]. To remove differences regarding rotation and spatial position, a Procrustes registration was applied based on the MT-BL coordinates. The registration was restricted to the MT-BL as a known structure. Following the registration, a shape model was computed using Principal Component Analysis [54,55,56].

### 2.6. Reconstruction of the MG-BL

The SSM was used to estimate the missing MG-BL on digitized plaster casts. To avoid statistical self-inference, a LOOCV scheme was applied: for each patient, an SSM was computed from the remaining data (excluding the investigated case) and was used to predict the patient’s mucogingival line. The prediction was performed by computing the posterior mean based on the available MT-BL [56].

### 2.7. Accuracy Analysis

The distance between each point in the actual MG-BL (as detected in the IOS) and the closest counterpart on the predicted MG-BL (as calculated by the SSM) was determined to estimate the prediction error. As the error distribution was non-gaussian, reporting the error as mean and accompanying standard deviation was not applicable. To obtain an idea about the distribution of the error, the median, as well as the 10th and 90th percentile from the pooled error values, were computed. 10th and 90th percentile were given in brackets following the median value.

## 3. Results

**Figure 3:**
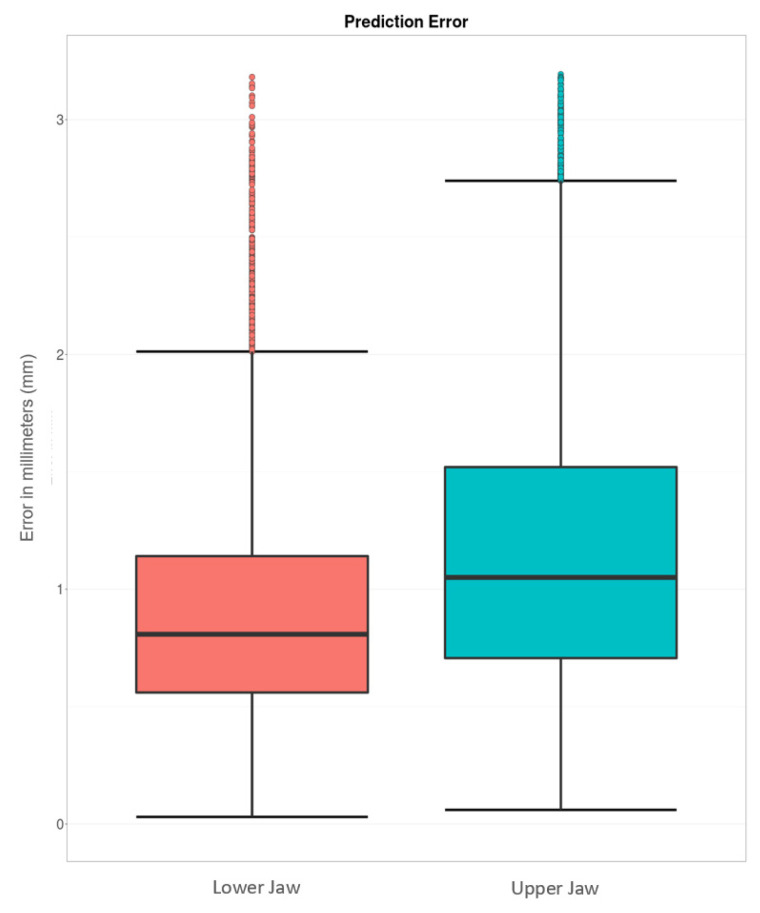
Boxplot showing the error distribution of the reconstruction of the MG-BL on all tested individuals using the developed SSM.

Eighty-two participants volunteered for study implementation. A total of 74 individuals were included in the study. Eight patients were excluded due to missing teeth. Of the 74 included individuals, 73 IOS of the maxilla and 71 IOS of the mandible were used for further examination. The analyzed IOS included the surrounding mucous membrane including colored texture information. Four scans were not analyzed because they were afflicted by mirroring artifacts. The age of the study participants was between 22 and 55 years with a mean age of 25.5 ± 4.4 years. Digital evaluation and accuracy analysis could be conducted successfully with the mentioned methods (see Figure 3).

### 3.1. Maxilla

**Figure 4:**
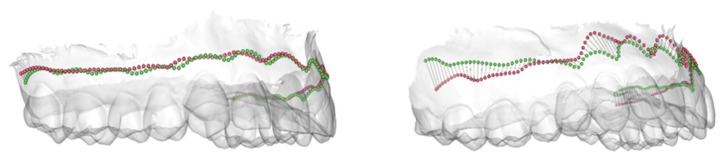
Best (**left**) and worst (**right**) prediction of the MG-BL of the maxilla based on the average error.

The median error for MG-BL reconstruction of the maxilla was 1.06 mm (0.49 mm–2.15 mm) (see boxplot in Figure 3). Figure 4 depicts the best (left side) and worst (right side) prediction of MG-BL.

### 3.2. Mandible

**Figure 5:**
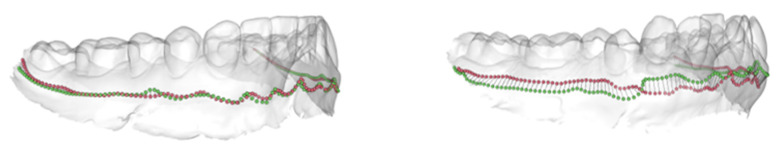
Best (**left**) and worst (**right**) prediction of the MG-BL of the mandible based on the average error.

The median error for MG-BL reconstruction of the mandible was 0.81 mm (0.38 mm–1.54 mm) (see boxplot in Figure 3). Figure 5 depicts the best (left side) and worst (right side) prediction of MG-BL.

## 4. Discussion

In this study, reconstruction of the MG-BL on digitized plaster casts was successfully performed using an SSM. With a median reconstruction accuracy of 0.81 mm for the lower jaw and 1.06 mm for the upper jaw, this workflow can be considered as an accurate reconstruction method. The generated SSM contains the shape information of two anatomical structures of 74 healthy individuals: the MT-BL and the MG-BL, which were extracted from IOS. Therefore, the reconstruction implies a strong covariation between the shape of the MT-BL and the MG-BL to successfully perform a reconstruction. Regarding the achieved accuracy of the proposed workflow, this assumption could be confirmed within the selected study group, even if the generation of the SSM was performed regardless of age or sex. As the evaluation was performed using an LOOCV, self-interference of the SSM with the investigated individual was avoided. This means that the digital scan of each plaster cast was presented to a temporary version of the deployed SSM like an unknown individual. Hence, the presented workflow manages to calculate the best possible representation of the MG-BL using an anatomical SSM, without requiring any information of the patient’s anatomy. This can be advantageous if only plaster casts are available, and the patient cannot attend further examination. Compared to other methods for depiction of the MG-BL [37,38] the presented workflow is fully digital and forgoes patient consultation and time-consuming and costly technical work. As digital platforms and artificial intelligence networks are increasingly used to obtain health-related information by both dental health-care professionals [57,58] and patients [59], this method addressed the upcoming trend of computerized health-care.

Historically, pre-operative planning of dental surgery was performed using physical models solely. Hereby all information required had to be depicted within or on top of the physical plaster cast to be used for planning purposes. Chee et al. presented an elaborate technique to fabricate master casts, which simulate the soft tissue dimensions using polyether impression material [60]. As this work was presented at a time when digital planning had not yet entered clinical routine, it was not designed to be fed into digital workflows. The manual steps required by this technique are a perfect example for the elaborate procedures that were necessary in the absence of computerized planning methods. Tarnow et al. [37] and Kaku et al. [38] presented methods for transferring MG-BL into virtual datasets, about twenty years later, when digital implant planning was already established. Both workflows involve marking the attached gingiva in the patient’s mouth: Tarnow et al. transfer the MG-BL to a plaster cast using an indelible pencil mark and subsequently fabricate a radiographic stent, which indicates the course of the MG-BL [37]. Kaku et al. mark the attached gingiva with radio-opaque sealer, which is then exposed during a cone-beam computed tomography (CBCT) scan [38]. While both authors report successful transfer of the MG-BL into virtual datasets, no accuracy analysis was presented. Both techniques come along with a time-consuming patient examination and a remarkable amount of technical work to be done before the MG-BL can be depicted digitally. Our workflow only requires the digital annotation of the MT-BL to calculate the missing information about the MG-BL and does not require a dental technician. Future research efforts can facilitate the procedure even further by automatizing the setting of landmarks using algorithmic support.

However, it must be noted that the presented workflow comes with several limitations. First of all, the SSM only calculates an estimation of the MG-BL, which may never be fully identical to the actual MG-BL of the patient. Even if the reported accuracy of this workflow suggests acceptable results within the selected study group, peculiarities of patient-specific anatomy, dental health and a multitude of other factors must be considered regarding their impact on the shape of the MG-BL. Recent publications presented a correlation between age and sex with gingival biotype [61,62]. Possible interference of patient specific features with the MG-BL are therefore likely, but not considered in the current version of the SSM yet. Moreover, the current version of the SSM does not include data about edentulous or partially edentulous patients. As tooth loss affects alterations in soft tissue dimensions [63], the data of (partially) edentulous jaws should be captured in an advanced version of the SSM to create a useful tool for implant planning. To enhance the SSM, patient specific features such as age, sex or ethnicity should be considered to take their possible effects on soft tissue dimensions into account. In this context, it must be noted that data curation may be possible in high numbers among healthy individuals, but those patients who may benefit the most from soft tissue reconstruction prior to implant planning are rare. This may lead to underrepresentation of the according anatomical shapes within the SSM and poor results for patients with unusual oral anatomy, such as clefts or syndromal malformations. The large amount of data required and the difficulties in estimating the learning success of artificial intelligence are a frequently mentioned barrier to the development of algorithm-based applications in medicine [64,65,66]. In this feasibility study, the reconstruction of the MG-BL was performed within a study group consisting of healthy individuals only.

To date, there is no study that describes the calculation of missing periodontal soft-tissue information in a digital dataset. Unlike available methods, the SSM can augment incomplete information by using empirical anatomical data without additional patient consultation. In clinical scenarios, this may be especially useful when plaster casts are scanned for planning purposes or IOS were performed insufficiently in the area of the MG-BL. As SSM-based reconstruction methods were recently reported as a viable way for computing digital wax-ups in a digital implant planning workflow [53], this study presents a relevant extension concerning relevant soft-tissue dimensions. In combination, the SSM could calculate important prosthodontic (e.g., dental wax-up of missing teeth) and periodontal information (e.g., supply of attached gingiva), which is necessary for proper implant planning. To gain clinical applicability, the inclusion of a high number of training data sets, which depict a multitude of different intra-oral situations, would be necessary. Although further work is required to extend the applicability of the SSM on (partially) edentulous patients, the rapid and feasible functionality of SSM makes it an effective digital method, which may save time and costs in future implant planning applications.

## 5. Conclusions

As the attached gingiva has a crucial role for the long-term success of dental implants, it should be considered in dental implant planning. Plaster casts are frequently used for implant planning but do not depict the MG-BL properly. Thus, the supply of attached gingiva on the alveolar ridge cannot be determined based on this data. In this study, a novel method for the depiction of the MG-BL in implant planning datasets was described. In contrast to methods described previously, no additional patient examination or elaborate technical work is necessary for this purpose. By implementing various anatomical datasets in an SSM, a digital reconstruction of the MG-BL can be performed by simply placing digital landmarks along the MT-BL. Using this method, the course of the MG-BL can be calculated in a fast and viable way during a planning session displaying the amount of attached gingiva. As the empirical knowledge of the current version of the SSM does not consider the multitude of different anatomical shapes among dental implant patients yet, further implementation of datasets is necessary to obtain clinical applicability of the presented workflow.

## Data Availability

The data presented in this study are available on request from the corresponding author. The data are not publicly available due to ethical restrictions.

## Abbreviations

CBCT	cone-beam computed tomography
FDI	Fédération Dentaire Internationale
IOS	Intraoral scanner
LOOCV	Leaving-One-Out Cross Validation
MG-BL	mucogingival borderline
MT-BL	mucous–tooth borderline
PLY	Polygon File
SSM	Statistical Shape Model
